# Perineural Spread and Base of Skull Involvement in Cutaneous Squamous Cell Carcinoma—A Critical Review from an Endemic Region

**DOI:** 10.3390/curroncol33050250

**Published:** 2026-04-27

**Authors:** Charles Y. Lin, Rahul Ladwa, Ryan Sommerville

**Affiliations:** 1 Department of Radiation Oncology, Cancer Care Services, Royal Brisbane and Women’s Hospital, Herston, QLD 4006, Australia; 2 Skull Base Unit, Royal Brisbane and Women’s Hospital, Herston, QLD 4006, Australia; ryan.sommerville@health.qld.gov.au; 3 School of Medicine, University of Queensland, Brisbane, QLD 4072, Australia; rahul.ladwa@health.qld.gov.au; 4 Department of Medical Oncology, Princess Alexandra Hospital, Woolloongabba, QLD 4102, Australia; 5 Department of Otolaryngology, Head and Neck Surgery, Royal Brisbane and Women’s Hospital, Herston, QLD 4006, Australia

**Keywords:** squamous cell carcinoma, head and neck, perineural spread, radiotherapy, skull base surgery

## Abstract

Perineural spread (PNS) from cutaneous squamous cell carcinoma (cSCC) to the skull base is increasingly recognised as a route of cancer spread. Management historically involved definitive radiotherapy to treat PNS at the skull base. In the endemic region with modern magnetic resonance neurogram (MRN) and skull base surgical expertise, the outcome has improved over the years. With the advent of immunotherapy, the outcome may be maintained while preserving critical organs in the head and neck region. We conducted a critical review of the literature to establish the treatment outcomes and pattern of failure as practice evolved. Furthermore, we described our skull base surgical and radiotherapy management guideline and outlined the emerging paradigm shift in the immunotherapy era.

## Simple Summary

This critical review summarised the evolving treatment paradigm of perineural spread (PNS) from head and neck cutaneous squamous cell carcinoma (cSCC) in the endemic area over the last 20 years. The treatment outcome has improved with the advances in medical imaging and radiotherapy technology, and the incorporation of skull base neurectomy. The current curative intent standard of care for this condition should routinely include radiotherapy. The emerging role of immunotherapy has provided a novel modality that may spare patients from the morbidities of upfront surgery and radiotherapy with clinical data accumulating.

## 1. Background/Epidemiology

When malignant cells invade the space around a large named nerve, it is referred to as perineural spread (PNS) [1]. Most frequently, tumours undergo retrograde spread towards the skull base; however, antegrade spread distally along the nerve can also occur [2]. PNS of cSCC is associated with significant morbidity, higher rates of local recurrence and poor overall survival. This is in comparison to perineural infiltration (PNI), which only involves small nerves and does not cause symptoms, and is not evident on medical imaging [3,4,5]. Perineural skip lesions have been described, but this phenomenon has subsequently been refuted and attributed to misinterpretation of Moh’s procedure and processing artefacts [1,6,7].

Bask et al. [3] reports the incidence of PNS with cSCC as <5%; while Balch et al. reports a range between 2 and 14% [8]. cSCC is the most treated head and neck malignancy with PNS in Australia, where 2 out of 3 people will be diagnosed with a skin cancer by the age of 70 [9].

PNS from cSCC affects more men than women. The average age at diagnosis was 66 years and the majority of patients gave a history of skin cancer (reflecting significant previous sun exposure) [10]. Often, the previous cSCC was associated with incidental PNI. However, the diagnosis of PNS should always be considered even in the absence of previous cSCC, as the skin SCC may have spontaneously regressed (unknown primary) but can still undergo perineural spread. In addition, patients can present with PNS from cutaneous SCC without microscopically identifiable small-nerve PNI [6]. The majority of patients presenting with PNS were immunocompetent [11,12,13].

## 2. Presentation and Diagnosis

Malignant PNS can be confused with other benign causes of cranial nerve dysfunction, such as viral neuritis, Bell’s palsy, or Trigeminal neuralgia [14]. Adding complexity to reaching a diagnosis of PNS is the finding that patients with PNS can have no known primary tumour, history of skin cancer, and/or incidental PNI in the primary [10]. Carroll et al. reported 20% of biopsy proven PNS from cSCC did not have an identifiable skin primary [15].

A progressive facial palsy and/or Trigeminal nerve dysesthesia are common presenting symptoms. Single-nerve disease was more common in de novo presentations, while 25% of the patients can present with synchronous multiple cranial nerve involvement [15]. The maxillary branch of the Trigeminal nerve (V2) was the most frequent nerve affected, followed by the ophthalmic branch (V1) [10,15]. The average maximum diameter of involved cranial nerve was 2.86 mm [10]. The majority of patients presented without regional nodal involvement [10,15].

### 2.1. Zonal Classification—William’s Classification and the XXXX Sub-Classification of Zone 2 Disease

An established cranial nerve zonal classification system was described by Williams et al. as shown in Table 1 [16]. The Williams classification is based on preoperative magnetic resonance imaging (MRI) and is a valuable tool in defining the extent of PNS and has been shown to correlate to prognosis [15,17,18,19].

The Royal Brisbane and Women’s Hospital (RBWH) subdivided Willam’s zone 2 into zone 2a and 2b. Zone 2a includes disease at the skull base foramen and extending proximally to, but not including the associated nerve’s ganglion (Table 1) [15]. Zone 2b includes disease associated with the nerve’s ganglion but not including the preganglionic segment. This subdivision further aids with surgical planning, which dictates surgical specialties required, and whether an intensive care unit (ICU) bed is required post-operatively. Details of surgical management will be discussed in the subsequent section.

As the RBWH classification system was only introduced in 2025, all the studies included in this review article used William’s zonal classification except Carroll et al. [15] Similarly, all the outcome data presented in this review article is based on the William’s classification unless specified otherwise.

### 2.2. Accuracy of MR Neurogram—Correlation with Histology

With the advent of 3-Tesla (3T) MRI neurography (MRN), significant advances in the ability to detect and define head and neck PNS of facial and Trigeminal nerves has been observed, particularly in relation to the involvement of superficial nerve branches. Radiological diagnosis of PNS was performed through asymmetrical nerve thickening or enhancement extending away from the primary tumour (or location of previous likely primary), infiltration of juxta-foraminal fat pads, and/or denervation changes in the muscles of facial expression or mastication [20].

Baulch et al. reported the correlation of 38 resected nerves to preoperative 3T MRN [8]. The author reported a sensitivity of 95% and a specificity of 84%, detecting PNS in 36 of 38 nerves and correctly identifying uninvolved nerves in 16 of 19 cases. They correctly identified the zonal extent of spread in 32 of 36 cases (89%). This study, however, included PNS from other non-cSCC histology. The author reported a tendency to under-call the zonal extent due to microscopic, radiologically occult involvement. Superficial large-nerve involvement also remains a difficult area of detection for radiologists and should be included as a ‘check area’ for routine assessment.

Schachtel et al. reported 38 cases of clinical PNS of the facial nerve from exclusively cSCC and correlated the preoperative MRN to the resected nerve histology [21]. They reported that 3T MRN had an 89% sensitivity in detecting VII PNS from cSCC, and a positive predictive value of 97%. When both imaging and histopathology were positive, 3T MRN was 100% accurate in assessing the zonal extent of facial-nerve PNS. Of note, incidental Trigeminal-nerve PNS was synchronously identified in 79% of the cases, with the auriculotemporal nerve (ATN) most commonly detected. Carroll et al. reported a MRN sensitivity of 91% based on a total of 82/90 histologically proven nerves correctly identified on preoperative MRN [15]. All 90 nerves had biopsy-/histology-proven PNS from cSCC. Pooled analysis of 259 patients with head and neck cancer by Sharma et al. reported an 89% sensitivity and 83% specificity with contrast-enhanced MRI [22].

3T MRN is the gold standard in preoperative imaging modality for PNS. Detection of peripheral PNS remains challenging, however, given the smaller nerve calibres, and is often the source of false negative reports [8,20]. Newer imaging sequencing and modalities such as black-blood MRI, positron emission tomography (PET)-MRI, and the use of tumour-specific radionuclide agents may also be of benefit, particularly given the known variability that exists between patients in the anatomical distributions of facial nerve branches [20,23].

## 3. Role of Skull Base Surgery

### 3.1. Extent of Surgery

The primary goal of surgery remains the achievement of clear resection margins. The morbidity associated with surgical intervention must always be weighed, particularly in elderly or comorbid patients or when critical structures are at risk. Intraoperative assessment via frozen section pathology is mandatory, with subsequent formal histopathological evaluation to ensure margin clearance.

### 3.2. Trigeminal Nerve

#### 3.2.1. V1 PNS

**Figure 1:**
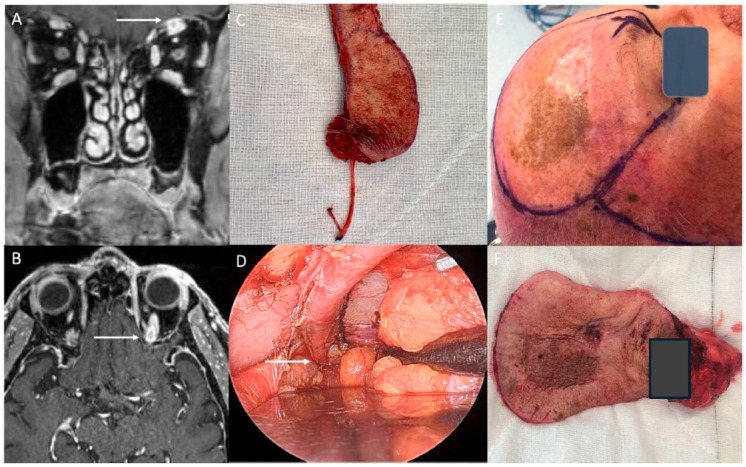
Left V1 nerve resection: (**A**,**B**)—magnetic resonance neurogram T1 FS + contrast left V1 zone 2a disease (white arrows), (**C**)—Right V1 nerve excision + eyebrow and skin lesion for zone 1 disease orbit sparing resection, (**D**)—Right V1 nerve shown to orbital apex (white arrow) for zone 1 disease resection, and (**E**,**F**)—Right V1 nerve + dermatomal excision (including orbital exenteration) and resultant specimen for zone 2a disease resection.

For zone 1 PNS, a transorbital, orbit-sparing approach (Figure 1) allows for resection of the supratrochlear and supraorbital nerves converging into the frontal nerve up to approximately 1 cm from the superior orbital fissure (SOF). The skin incision is determined by the cutaneous lesion location or may involve a cosmetically favourable eyebrow incision to access the supraorbital notch and superior orbital tunnel. In cases of zone 2a or 2b involvement of the V1 nerve, orbital exenteration combined with lateral orbitotomy and pterional craniotomy is necessary. A temporalis muscle split, with or without zygomatic osteotomy, facilitates lower pivot access to the proximal ganglion (see below).

#### 3.2.2. V2 PNS

**Figure 2:**
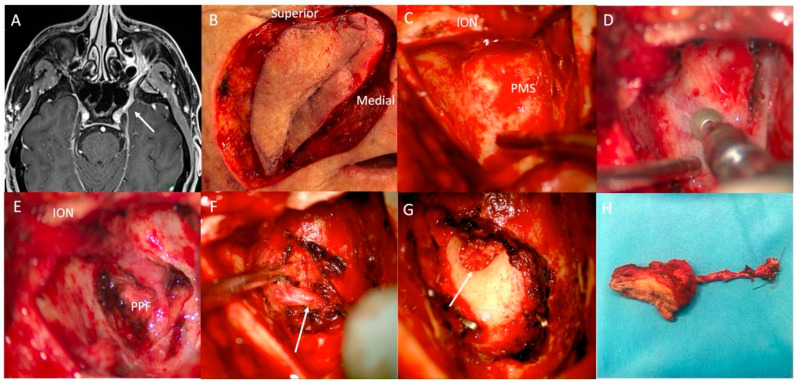
Left V2 nerve resection: (**A**)—Left V2 zone 2b disease on magnetic resonance neurogram T1 FS + contrast (arrow at Trigeminal ganglion). (**B**)—Right cheek skin lesion excision. (**C**)—Anterior maxillary window performed with infra-orbital nerve (ION) remaining pedicled at top of field and posterior maxillary sinus wall visible at deep aspect. (**D**)—Removal of posterior maxillary sinus bony wall using diamond drill. (**E**)—pterygopalatine fossa (PPF) contents exposed with ION seen running from attachment to skin lesion to superior aspect of PPF. (**F**)—Maxillary artery (arrow) isolated for ligation. (**G**)—Skin lesion, ION and PPF removed to Foramen rotundum (arrow) prior to drillout of pterygoid base to allow intracranial nerve dissection. (**H**)—Resected skin lesion with ION emerging from deep aspect of specimen and stitched at proximal end for pathological notation.

Zones 1 and 2a V2 PNS require a transfacial, transpterygoid approach, as described by RBWH and also illustrated in (Figure 2) [15]. Residual or primary cutaneous lesions necessitate wide local excision, which may provide open access. Alternatively, a Weber Ferguson incision with lip split, and occasionally lower lid extension, is employed. Note that any lower eyelid extensions increase the risk of an ectropion. Extension of the access via a sublabial mucosal incision and subperiosteal dissection expose the infraorbital nerve (ION) at its foramen. Anterior maxillotomy using a sagittal saw and osteotomies provides maximal surgical access while preserving the orbital rim and piriform aperture for structural stability and cosmesis, while respecting the roots of the adjacent teeth. The ION is released inferiorly from its canal without breaching the periorbita. The posterior maxillary wall is removed with a diamond drill and Kerrison rongeurs, exposing the pterygopalatine fossa (PPF). Key structures such as the maxillary artery, posterior superior alveolar nerve, descending palatine nerve, sphenopalatine artery, and Vidian nerve are identified and managed in that order. This allows vessel control, en bloc dissection of the PPF contents and margin control of any antegrade nerve spread. The ION and entire PPF contents are mobilised in continuity to the foramen rotundum for frozen section analysis. Additional drilling of the foramen rotundum using a transpterygoid exposure may result in further proximal margin control for V2, while avoiding injury to middle fossa dura and the petrous internal carotid artery [24]. If necessary, V3 dissection can extend via removal of the base of the pterygoid, following the nerve into the infratemporal fossa and then proximally to its junction with V2 at the Trigeminal ganglion. The extent of transnasal intracranial V2 dissection beyond foramen rotundum depends on disease extent, surgical access, bleeding control, and dural mobility, with positive margins on frozen section necessitating a craniotomy. Endoscopic trans-nasal approaches alone are generally insufficient for zone 1 and zone 2a V2 PNS, especially when cutaneous or premaxillary involvement exists, although endoscopes may assist in certain intracranial or infratemporal dissection aspects. Zone 2b V2 PNS requires a similar transfacial, transpterygoid approach combined with a craniotomy to access the Trigeminal ganglion (see below).

#### 3.2.3. V3 PNS

For zone 1 and 2a V3 PNS, disease often tracks via the ATN, requiring total parotidectomy (conservative or radical, depending on facial nerve involvement) and a preauricular infratemporal fossa (ITF) approach with ascending mandibulotomy or temporomandibular joint capsule resection to access the foramen ovale [25]. A small temporal craniotomy may facilitate extension to the Trigeminal ganglion. Retrograde spread from V2 to the Trigeminal ganglion may then spread anterogradely to secondarily involve V3. In such cases, a transfacial anterior approach with pterygoid base drillout and pterygoid muscle release from the lateral pterygoid plate may be employed for short-segment V3 exposure, though access to the multiple branches of V3 in the ITF is limited via this method and may require lateral access via a true ITF approach [24,25].

### 3.3. Facial Nerve PNS

**Figure 3:**
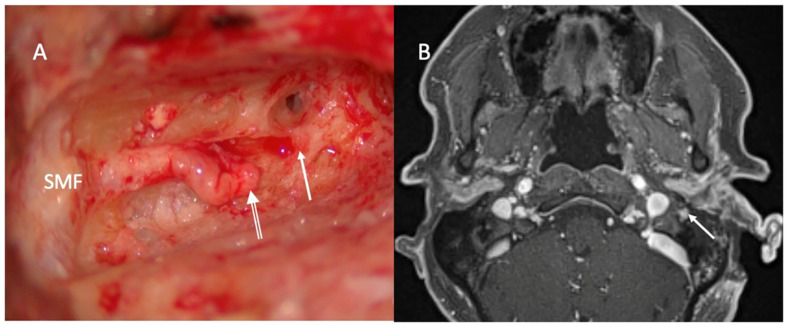
Left VII zone 2a nerve resection. (**A**)—Surgical photo of cut left facial nerve (double arrow) resected at the 2nd genu (white arrow) prior to frozen section analysis of the proximal nerve end. (**B**)—Axial MRN T1 FS + contrast showing left facial nerve zone 2a PNS with enhancement of the nerve in the mastoid portion (white arrow). Abbreviations: PNS: Perineural spread; SMF: stylomastoid foramen; and MRN: Magnetic resonance neurogram.

Zone 1 facial nerve PNS (Figure 3) is addressed via standard radical parotidectomy, with frozen section confirmation of clear margins at both the stylomastoid foramen and distal branch nerve endings. Zone 2a disease extends from the stylomastoid foramen to but not including the geniculate ganglion. If the external auditory canal (EAC) is not involved, a canal wall up mastoidectomy with posterior tympanotomy is often sufficient for access to the facial nerve up to the 2nd genu. Extensions may include radical mastoidectomy with blind sac closure of the EAC or formal lateral temporal bone resection for tympanic facial nerve segment access and EAC disease control as required. The facial nerve is traced as required to the second genu or tympanic segment based on preoperative MRN findings, with frozen section analysis at these points. Zone 2b facial nerve PNS involves the geniculate ganglion, while zone 3 disease affects the internal auditory canal (IAC) segment. Both require a full translabyrinthine resection to access the nerve through the ganglion, labyrinthine canal, and IAC. Disease extending to the brain stem is unresectable, and surgery for zone 3 involvement should only proceed if a clear margin remains achievable in the IAC segment of the nerve.

### 3.4. Craniotomy for Trigeminal Ganglion Dissection

Complete access to the Trigeminal ganglion can be attained via a standard pterional craniotomy. The craniotomy is extended to the root of the zygoma, permitting a lateral extradural approach along the middle fossa. This is combined with an anterolateral approach, requiring drilling of the lateral sphenoid wing and then division of the meningoorbital fold (at the lateral aspect of the SOF). Appropriate division of this fold facilitates entry into the inter-dural plane. Development of this plane facilitates exposure of the inner layer of the lateral wall of the cavernous sinus, along with the Trigeminal ganglion and its proximal divisions. In cases requiring targeted access to V1, removal of the lateral orbital wall may provide direct exposure to the SOF and allow the continuation of dissection from the SOF to the Trigeminal ganglion. In cases where exposure of only the inferior portion of the ganglion is required (e.g., V2 and V3), a mini-pterional craniotomy can be employed, with splitting of the temporalis facilitating a lateral extradural approach to the foramen rotundum and ovale. The involved branch of the Trigeminal nerve is sectioned as it meets the ganglion with frozen section analysis at this point. If a positive margin is attained, further resection of the Trigeminal ganglion occurs, though with careful consideration of the effects of complete Trigeminal nerve section.

### 3.5. Reconstruction

Reconstructive procedures are tailored to the extent of soft tissue and bony resection performed, ensuring both functional and cosmetic restoration based on the individual surgical defect. Additional goals of reconstruction include repairing any intraoperative cranial spinal fluid leak and ensuring adequate soft tissue coverage of internal carotid artery. This may require local skin rotational flaps or pedicled flaps if more significant defects result from the resection. Allowances need to be made for the atrophic changes that will occur with post-operative radiation.

## 4. Role of Radiotherapy

The principal aims of definitive or adjuvant radiotherapy (RT) are to prevent cutaneous relapse, extracranial skull base recurrence, and central intracranial control [2]. There are generally four dose levels of clinical target volume (CTV). Dose levels are expressed in equivalent doses in 2-Gy fractions (EQD2). CTV70 (EQD2 70 Gy) includes gross disease (based on preoperative MRI/CT/PET) plus a 5 mm margin but cropped to anatomical barriers. CTV70 is used for patients who did not have surgery to remove macroscopic disease either at the skin primary site and/or involved nerve and/or lymph node(s). CTV66 (EQD2 66 Gy) includes the surgical bed (skin and/or nerve, and/or nodes) where margins are microscopically involved. A 10 mm margin should be considered in patients with involved surgical margins at the skin/dermis site. CTV 60 (EQD2 60 Gy) includes the surgical bed where margins are microscopically clear. CTV50 (EQD2 50 Gy) includes the at-risk elective area outside the CTV60/66. It also encompasses the nerve zone proximal to the nerve resection, and/or the nodal station adjacent to the dissected nodal basin felt to be at risk. The cutaneous distribution of the named nerve (dermatome) should be included in CTV50 while respecting adjacent critical organs. Organs at risk include the globes, lacrimal glands, optic nerves, chiasm, brainstem and brain. Our standard margin expansion from CTV to the planned target volume (PTV) is 5 mm. Bolus should be applied to the local surgical bed to ensure adequate skin dose delivery. Preoperative MRN should be fused to the planning CT to define the tumour bed and contours peer-reviewed by an experienced skull base radiation oncologist. All treatment should be delivered via intensity modulated radiation therapy (IMRT). In cases where the nerve margins are involved, the skull base surgeon(s) should attend the planning room to advise on the location of the positive PNS margin(s). This recommended CTV delineation is summarised in Table 2.

**Figure 4:**
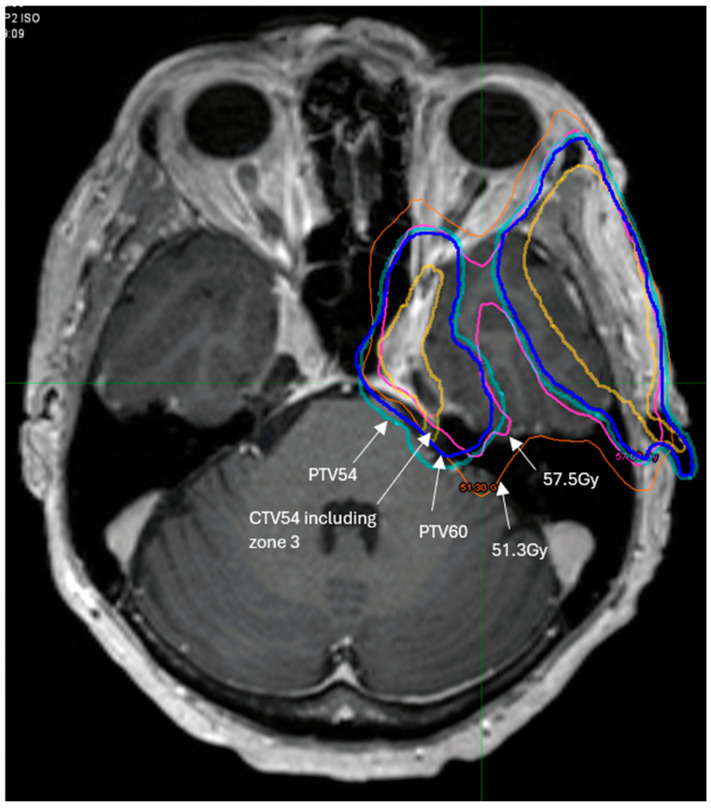
A patient with left V2 zone 2b disease resected with clear margin. Preoperative magnetic resonance neurogram fused with planning CT. PTV 60 (dark blue) encompassed the resected peripheral perineural disease inclusive of the cutaneous primary site on the left cheek (flap repaired) and V2 nerve tract to and including the Trigeminal ganglion. The PTV54 (cyan) extended to the cisternal segment of the left Trigeminal nerve (zone 3). Note the 95% isodose line for PV60 = 57.5 Gy (pink) was prioritised for brainstem constraint (Dmax limit ≤ 54 Gy) over coverage of PTV.

Examples of radiotherapy contour volume and doses are illustrated in Figure 4. Due to the low incidence of failure in zones 2 and 3, we allow compromise of PTVs in the proximity of critical organs at risk, e.g., brainstem (Dmax ≤ 54 Gy) and optic chiasm (Dmax ≤ 54 Gy).

## 5. Treatment Outcomes

Prior to the incorporation of skull base surgery (SBS), patients were treated with wide local resection of involved soft tissue with macroscopic positive nerve margins, and post-operative radiation therapy (PORT). This yielded a five-year relapse-free survival (RFS) in the order of 20–50% (Table 3) [26,27,28,29,30]. Limiting factors in the interpretation of these studies include variable dose fractionation schedules (50–74.9 Gy utilised), and some patients were treated on a bi-daily regimen. Furthermore, non-standardised extent of RT to the skull base also confounded the interpretation making cross-trial comparisons challenging. Most of these patients were treated with either 2-dimensional or 3-dimensional RT but not IMRT. It should also be noted that 3T MRN was not routinely performed in these studies and most studies also combined basal cell carcinoma with SCC in the outcome analysis.

The outcome of PNS associated with cSCC has improved with the combination of SBS and PORT, and the results are summarised in Table 4, mainly from two high-volume quaternary institutions in Queensland, Australia, of highly comparable epidemiology. The reported 5-year RFS was in the order of 60–65%, 5-year disease-specific survival of 68–77%, and 5-year overall survival of 58–67% [15,17,18,19]. These studies included only SCC, and all had preoperative MRN assessment. Of note, the majority but not all patients (82.4% Schachtel et al.; 76% Crawford et al.; 85% Carroll et al.) were treated with combined SRS and adjuvant IMRT, except the study reported by Warren et al., in which all patients received the full combined modality treatment [15,17,18,19].

In contrast to definitive RT alone, the SBS data do appear to show superior outcome with an improvement of RFS in the order of 20–25%. The study by Crawford et al. reported a significantly worse outcome if patients were treated with single-modality approach (SBS alone or RT alone) [19], while Carroll et al. reported a trend, though not statistically significant, of inferior outcome in patients treated with a single-modality approach [15].

We have summarised the published oncological outcome of patients with head and neck skin cancer treated prior to SBS (Supplementary Material S1), while Table 3 summarised the published outcome treated with combined SBS and PORT.

## 6. Pattern of Failure and the Importance of Local Skin/Dermal Sites

As shown in Table 5, the local skin/dermal site is the most common site of recurrence as reported by multiple studies [15,17,18,19,27,28]. This highlighted the possible undertreatment of the index skin site and adjacent dermis. As nerves travel distally towards the skin, the calibre diminishes, making it more difficult to detect on MRN, which may also lead to undertreatment of the skin/dermis in terms of radiotherapy dose and extent. Due to the retrospective nature of the studies, it is difficult to ascertain whether the skin/dermal recurrence was in-field, at the edge, or out-field, and whether bolus was applied to the recurrent sites.

Strategies to reduce the high prevalence of local skin/dermal failure include wider surgical excision and considering dermatomal (cutaneous distribution of the named nerve) resection in patients with extensive multifocal perineural infiltrations at the index skin site. From the radiotherapy perspective, a wider margin (CTV66 = cutaneous surgical bed and gross disease on preoperative imaging + 1 cm) should be considered for patients with involved skin or PNI surgical margins. Patients with extensive multifocal PNI at the skin site may also be considered for dermatomal (cutaneous distribution of the named nerve) irradiation. Bolus should be applied to ensure the skin/dermis of the surgical bed consistently receive the full dose.

## 7. Prognostic Factors

For patients treated with combined SBS and PORT, several prognostic indicators were identified.

1. William’s Zone 1 disease was consistently found to have better RFS than zone 2 and zone 3 diseases [15,18,19,20]. This applies to treatment with or without SBS. RBWH zone 2b disease did not show worse outcomes than zone 2a lesions, though more extensive surgery with a craniotomy was required to maintain those outcomes.
2. Multiple cranial nerve involvement was shown to be a poor prognostic factor with or without SBS [15]. This is supported by Schachtel et al. who reported poorer outcomes when Trigeminal and facial nerves were synchronously involved [18].
3. Incomplete resection of the skin/dermal disease or PNS was shown to be a poor prognostic factor in Warren et al. although the difference did not reach statistical significance [17]. The difference was not evident in Carroll et al., but Schachtel et al. showed a statistically significant detriment in those with involved surgical margins [15,18]. Based on the first principle, resection with a clear margin should always be the aim as it reduces tumour bulk and accurately defines the extent of the disease.

## 8. Immunotherapy in PNS Associated with cSCC

The management of advanced large-nerve PNS in cSCC is challenging, with the proximity to vital head and neck anatomy requiring technically demanding surgery and often resulting in substantial treatment-related morbidity, including loss of function in involved cranial nerves.

Prior to the introduction of immune checkpoint inhibitors (ICIs), the prognosis was poor for these patients, with limited systemic therapeutic options available and no agreed standard of care. Retrospective analyses have shown that cytotoxic chemotherapy—such as platinum-based agents, taxanes and fluoropyrimidines—can exhibit clinical activity, but responses are short-lived, with safety being a primary concern for this typically older, comorbid population [31]. Studies of epidermal growth factor receptor (EGFR) inhibitors demonstrate a more tolerable toxicity profile but similarly show modest benefit [32]. Clinical trials using immune checkpoint inhibitors (ICIs) targeting the programmed cell death-1 (PD-1) axis have demonstrated high and durable responses in recurrent/metastatic or locally advanced cSCC for which no curative local treatment options exist [33,34,35]. The responsiveness has been attributed to the immunogenic nature of cSCC, which is driven by factors such as high tumour mutation burden, ultraviolet-induced genetic alterations, and overexpression of tumour antigens [35]. The final long-term analysis of the EMPOWER-CSCC-1 found an objective response rate of 47% and a median progression-free survival of 26 months in patients with metastatic and locally advanced, unresectable cSCC [35]. These findings have supported FDA approval for anti-PD-1 and anti-PD-L1 monotherapy in the treatment of locally advanced or metastatic cSCC [33,34,35,36,37].

Importantly, within these foundational studies for anti-PD-1 therapies, there is no exploration of the PNS subgroup, nor are their outcome data known. Although patients with PNS were not specifically excluded from prospective clinical trials, the ability to accurately measure the disease can be challenging from an eligibility perspective and may limit their inclusion. Retrospective studies involving both PNS at diagnosis and/or at recurrence have been published with encouraging results. Response rates among four studies, involving a total of 56 patients (10–20 patients from each study), range from 69 to 83% [38,39,40,41]. Response assessment to ICIs can be difficult to interpret, with a combination of neural enhanced MRI and clinical evaluation being utilised, with standard radiological assessment including RECIST1.1 providing less than optimal response evaluation [42].

The success of ICIs in trial-eligible populations has driven interest in evaluating outcomes among trial-ineligible groups. Advanced cSCC frequently occurs in patients who are excluded from clinical trials, such as those who are immunocompromised or have significant comorbidities and reduced performance status. Retrospective real-world studies have demonstrated the safety and efficacy of ICIs in these populations [43,44,45,46]. In a large Australian series in which 31% of patients were immunocompromised and 21% had an ECOG score of 2 or more, the estimated 12-month overall survival (OS) was 78% and PFS 65%, which compares favourably with the 81% and 53%, respectively, reported in the registrational cemiplimab clinical trial [43]. This was a retrospective study and was not specifically targeting patients with cSCC and PNS (the focus of this review) but any advanced cSCC. The study showed similar toxicities but poorer outcome for immune-suppressed patients treated with immunotherapy, but clinical benefit can still be derived. The authors concluded that immunotherapy could be beneficial for a broader range of patients with advanced cSCC than those eligible for participation in clinical trials.

More recently, cemiplimab following surgery and PORT for high-risk resectable cSCC has demonstrated a significant improvement in disease-free survival (DFS) in the C-POST study [47]. Out of a total of 415 patients, a DFS at 24 months of 64.1% was observed in those not receiving ICI, while the addition of 12 months of cemiplimab resulted in a significantly higher DFS of 87.1% in the C-POST study. Approximately 15% of the patients recruited in the C-POST study had evidence of PNS.

Neoadjuvant ICI has potential advantages, including: (1) reduction in surgical morbidity by reducing tumour burden; (2) provision of immediate information about response to ICI; and (3) access to treated tissue to provide insight into resistance mechanisms and to identify biomarkers of response. Consequently, the role of anti-PD-1 therapy has been investigated in the neoadjuvant setting to assess pathological response at the time of planned surgery where studies have shown a 40–55% complete (no residual viable tumour cells) pathological response (pCR) rate in patients with resected cSCC [48,49,50]. In the prospective, phase II multicentre study of 79 patients with stage II–IV(M0) resectable cSCC, patients received up to four cycles of cemiplimab before proceeding to surgery with 51% achieving a pCR and a 24-month event-free survival (EFS) of 86% [49]. Given the high rates of pCR and early promising event0free survival data outlined above, recent clinical trials have investigated whether surgery and/or PORT can be safely de-escalated following neoadjuvant ICI treatment. This is especially desirable in an elderly, comorbid population where there is significant potential morbidity associated with definitive management in predominantly head and neck cSCC.

**Figure 5:**
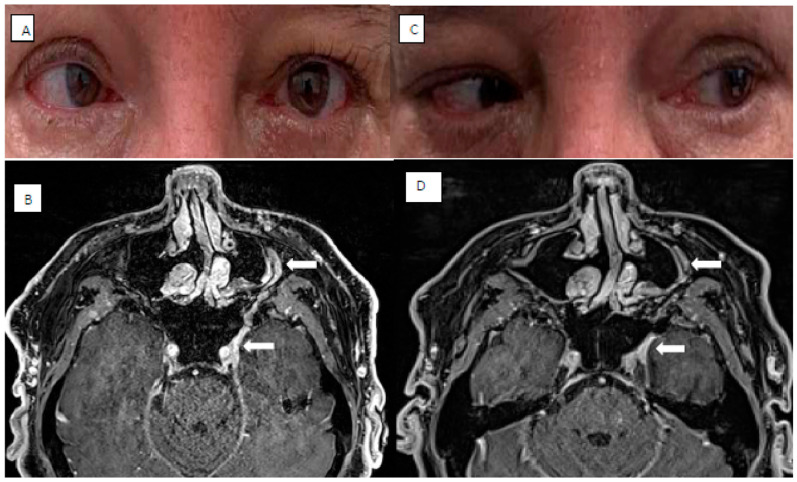
A man presented with a 6-month history of left forehead numbness, diplopia and reduced extraocular left eye movements on a background of resected facial cutaneous squamous cell carcinoma. On examination, there was reduced sensation over the left upper two-thirds of his face and ophthalmoplegia of the 3rd, 4th and 6th cranial nerves with normal optic nerve function (Panel **A**). A neural-enhanced MRI revealed enhancement of both the frontal branch of the ophthalmic division of the Trigeminal nerve (V1) extending back to the superior orbital fissure and the maxillary division of the Trigeminal nerve (V2) with atrophy of the superior oblique muscle consistent with perineural spread (Arrows; Panel **B**). The patient commenced Programmed Death Receptor-1 (PD-1) inhibitor with Cemiplimab. Following 3 months of treatment, his ophthalmoplegia improved on lateral gaze (Panel **C**) with regression of the neural involvement on MRI showing reduced perineural thickening and contrast enhancement (Arrows; Panel **D**).

Specifically, patients with PNS may have the opportunity to trial ICI with the potential for cranial nerve function recovery and symptomatic improvement without losing the opportunity for curative intent surgery and or radiotherapy (Figure 5).

The De-Squamate study was a multicentre phase II study, to assess the effectiveness of pembrolizumab and subsequent response-guided surgical and radiotherapy treatment de-escalation of resectable cSCC [51]. A clinical or pathological complete response was seen in 63% of participants avoiding curative intent radiotherapy and/or surgery with a 94% 12-month event-free survival. Patients with PNS were included in this study. Pembrolizumab led to a high rate of clinical complete response in resectable cSCC and demonstrated the potential to omit surgery and RT.

While the experience of treating resectable cSCC with PNS with neoadjuvant ICI remains limited, PORT should be offered to patients with residual disease in the absence of clinical trials. The dose and extent of PORT should be based on pre-ICI images as recommended in Table 2. For patients with a complete pathological response following neoadjuvant ICI, it is prudent to ensure all disease evident on PET and MRN was resected. If all the disease was confidently resected, considerations can be given to omit PORT provided patients are being followed up regularly with MRN and managed in a centre with skull base expertise. Future dedicated studies in patients with PNS are needed to better define the effectiveness of immune checkpoint inhibitors, particularly given the challenges in accurately assessing response with conventional imaging and the currently limited understanding of pathological response following immunotherapy. Such a study is currently enrolling in Queensland, Australia, specifically assessing the use of neoadjuvant ICI for patients with resectable zone 1 and 2 PNS. Patients will be offered upfront ICI for four cycles, followed by clinical and radiological assessment with subsequent response-adapted treatment.

## 9. Conclusions

The outcome of combined skull base surgery and PORT for cSCC with zone 1 and 2 PNS is highly acceptable. Radiotherapy should be routinely offered to patients treated with curative intent, unless in the setting of a clinical trial utilising immunotherapy. Given the high prevalence of local failure at skin/dermal sites, a clear skin surgical margin and wider RT margins at the skin/dermis should be considered. Adequate bolus to ensure the skin/dermis receive the full prescribed RT dose is also recommended. Medically unresectable zone 1 or 2 PNS or patient refusal to undergo SBS should be offered curative intent definitive RT or palliative immunotherapy. Zone 3 PNS has a poor outcome and should be offered palliative immunotherapy. The emerging role of neoadjuvant immunotherapy in cSCC may influence future management both in resectable or unresectable settings while more mature clinical data are awaited.

## Figures and Tables

**Table 1:** William’s zonal classification of cranial nerves perineural spread and the Royal Brisbane and Women’s Hospital (RBWH) modified zonal classification of Cranial Nerves.

**Zonal classification of cranial nerves (according to Williams et al.) [16]**				
**Cranial Nerve**	**Zone 1**	**Zone 2**	**Zone 3**	
V1	Peripheral nerve to superior orbital fissure	Superior orbital fissure to but not including Trigeminal ganglion cistern	Trigeminal ganglion cistern to brainstem	
V2	Peripheral nerve to foramen rotundum	Foramen rotundum to but not including Trigeminal ganglion cistern	Trigeminal ganglion cistern to brainstem	
V3	Peripheral nerve to foramen ovale	Foramen ovale to but not including Trigeminal ganglion cistern	Trigeminal ganglion cistern to brainstem	
VII	Peripheral nerve branches in facial and parotid region to stylomastoid foramen	Styloid mastoid foramen through labyrinthine segment up to the internal auditory canal	Internal auditory canal to brainstem	
**RBWH modified zonal classification of cranial nerves [15]**				
**Cranial Nerve**	**Zone 1**	**Zone 2a**	**Zone 2b**	**Zone 3**
V1	Peripheral nerve to superior orbital fissure	Superior orbital fissure to but not including Trigeminal ganglion	Trigeminal ganglion but not the preganglionic segment	Trigeminal ganglion cistern to brainstem
V2	Peripheral nerve to foramen rotundum	Foramen rotundum to but not including Trigeminal ganglion	Trigeminal but not the preganglionic segment	Trigeminal ganglion cistern to brainstem
V3	Peripheral nerve to foramen ovale	Foramen ovale to but not including Trigeminal ganglion	Trigeminal ganglion but not the preganglionic segment	Trigeminal ganglion cistern to brainstem
VII	Peripheral nerve branches of facial and parotid region to stylomastoid foramen	Stylomastoid foramen to tympanic segment of facial nerve	Geniculate ganglion involved	Internal auditory canal to brainstem

**Table 2:** Recommended radiation doses and definition of clinical target volumes.

Dose Level	Description	Nerve	Skin/Dermis	Nodal Region
**CTV70**	Macroscopic residual disease + 5 mm	The gross PNS + 5 mm	The gross skin/dermal disease + 5 mm	Gross involved nodes + 5 mm
**CTV66**	This volume should be utilised when surgical margins are microscopically involved with input from the surgeon(s) and pathologists. The preoperative volume based on the MR neurogram/PET/CT should be contoured	The preoperative PNS + 1 cm proximal to the nerve track: This would often include the adjacent nerve zone by default. E.g. Zone 2a PNS with involved proximal margin should have the gross preoperative PNS + zone 2b included in CTV66	The preoperative gross peripheral PNS and/or index skin disease and/or flap/skin graft should all be included + 1 cm margin	The preoperative nodal disease + 1 cm margin
**CTV60**	Surgical bed where margins were clear. This should include the neurectomy and/or skin primary sites	Neurectomy site + 5 mm	Skin excision site and flap/skin graft + 5 mm	Dissected nodal basin + 5 mm
**CTV50**	Elective volume outside the operative bed	The next zone above the respected PNS should be elective included in CTV50, e.g., PNS zone 2a with clear surgical margin should have zone 2b included.	The cutaneous distribution of the named nerve (dermatome) should be included in CTV50	Elective nodal dissection and irradiation is generally not recommended for cN0 disease. In pN+ disease, the next draining nodal echelon should be included in CTV50, e.g., parotid nodal SCC with CN VII PNS treated with parotidectomy (no upper neck dissection) should have ipsilateral level II and III electively irradiated

Abbreviations: CTV: Clinical target volume; PNS = Perineural spread.

**Table 3:** Oncological outcome—definitive radiotherapy without skull base surgery.

	Sample Size/ Histology	RT Dose	OS at 5 Years	DSS at 5 Years	DFS at 5 Years	RFS at 5 Years	Poor Prognosticators
Ballantyne et al. [26]	80; Mostly SCC	Not clearly described	-	-	-	46%	-
Bourne et al. [27]	13; SCC	50–55 Gy/20–25#	-	-	-	20%	-
Garcia Serra et al. [29]	76; SCC or BCC	Median dose: 70 Gy/39# in either once-daily or twice-daily fractionation. 2 dimensional RT planning	-	-	-	50%	-
Lin et al. [28]	44; SCC	Median dose 60 Gy in 30 fractions at 5 fractions/week.	-	-	-	39%	Multiple cranial nerves; V1 and/or V2 nerve PNS worse than V3 or facial nerve
Balamucki et al. [30]	65; SCC or BCC	Median dose: 74.9 GGy/39# or 70.2 Gy and interstitial implant; once daily or twice daily treatment	54%	64%	51%	-	Non-significant trend towards worse outcome for macroscopic/ central disease.

Abbreviations: SCC: Squamous cell carcinoma; PNS: Perineural spread; RT: Radiotherapy; #: fractions; OS: Overall survival; DSS: Disease-specific survival; DFS: Disease-free survival; and RFS: Relapse-free survival.

**Table 4:** Oncological outcome—skull base surgery and/or radiotherapy for cSCC with PNS.

	Sample Size, Description, Treatment	RT Dose	OS at 5 Years	DSS at 5 Years	DFS at 5 Years	RFS at 5 Years	Poor Prognosticators
Warren et al. [17]	50 patients, PNS to V and/or VII; All had combined SBS + PORT	50–63 Gy/25–30#	64%	75%	-	62%	A non-significant detriment toward worse OS for those with involved margins. Zones 2 and 3 PNS did worse than Zone 1.
Schachtel et al. [18]	73 patients, PNS to VII cranial nerve; 67% had concurrent V involvement; 65.8% had curative surgery, 82.4% of those had PORT	60 Gy/30#	58.1%	68.7%	50.7%	-	Nodal metastasis; involved margins Higher zonal extent; concurrent zone 2 of 5th cranial nerve PNS.
Crawford et al. [19]	53 patients, PNS to V1; 76% had combined S + RT with; 34 patients had orbital exenteration	59 Gy/29#	61.9% (Zone 1: 82% Zone 2: 52.5% Zone 3: 28.6%)	74.6%; (Zone 1: 94.1% Zone 2: 65.3% Zone 3: 42.9%)	62.1% (Zone 1: 82.5% Zone 2: 48.1% Zone 3: 28.6%)	-	Single-modality surgery or RT; higher William’s zone PNS
Carroll et al. [15]	80 patients, PNS to any cranial nerve; 85% had combined SBS + PORT	60–74 Gy/30–37#	67% (Zone 1: 67% Zone 2a: 59% Zone 2b: 76%)	77% (Zone 1: 78% Zone 2a: 74% Zone 2b: 73%)	-	61% (Zone 1: 73% Zone 2a: 53% Zone 2b: 35%)	Multiple cranial nerves involvement

Abbreviations: cSCC: Cutaneous squamous cell carcinoma; PNS: Perineural spread; RT: Radiotherapy; SBS: Skull base surgery; PORT: Postoperative radiotherapy; OS: Overall survival; DSS: Disease-specific survival; DFS: Disease-free survival; and RFS: Relapse-free survival.

**Table 5:** Pattern of relapse following treatment.

	Histology	Treatment	Patterns of Failure
Lin et al. [28]	SCC	RT alone	Local: 79% Nodal: 16%
Garcia-Serra et al. [29].	BCC + SCC	RT alone	90% at primary site (skin and/or perineural tract)
Warren et al. [17]	SCC	SBS +/− PORT	Local peripheral (skin) in-field recurrence: 72% Local central in-field (meninges/brainstem) recurrence: 6% Local central + peripheral in-field: 11% Local peripheral (skin) out-field: 33% Local in-field + regional out-field + distant metastasis: 6% Mean time to any recurrence was 24 months with 81% occurring within 4 years
Crawford et al. [19]	SCC	SBS and/or PORT	Local recurrence: 58% (mostly in-field) Central perineural recurrence: 33% (50% in-field) Distant recurrence: 8%
Schachtel et al. [18]	SCC	SBS and/or PORT	Local skin/subcutaneous tissues: 58% 82.4% locoregional, including recurrences peripherally in the skin or subcutaneous tissues (57.9%), Central PNS (26.3%), Regional nodes (5.3%). Distant recurrences: 21%
Carroll et al. [15]	SCC	SBS and/or PORT	Local cutaneous/dermal recurrence: 60% Central perineural recurrence: 21% (half of these had synchronous local relapse, i.e., 11% had isolated central perineural relapse) Distal perineural recurrence below the skull base: 25% (half of them had synchronous local relapse) Regional recurrence: 18% (3 cases also had synchronous local recurrence; 7% had isolated nodal recurrence); Distant recurrence: 18% (one case also had local recurrence; 14% had isolated distant relapse)

Abbreviations: SCC: Squamous cell carcinoma; BCC: Basal cell carcinoma; RT: Radiotherapy; PORT: Postoperative radiotherapy; and SBS: Skull base surgery.

## Data Availability

Research data are not available at this time.

## Supplementary Materials

The following supporting information can be downloaded at: https://www.mdpi.com/article/10.3390/curroncol33050250/s1, Supplementary Material S1: Oncological outcome—Definitive radiotherapy without skull base surgery.
