# Supplementary material for: Perineural Spread and Base of Skull Involvement in Cutaneous Squamous Cell Carcinoma—A Critical Review from an Endemic Region
Source: Curr Oncol. 2026 Apr 27;33(5):250. doi: 10.3390/curroncol33050250 (PMC13205704; doi:10.3390/curroncol33050250)
Supplement: Supplementary file 1 [file curroncol-33-00250-s001.zip › curroncol-4183814-supplementary.pdf]

Supplementary Material S1.

Oncological outcome- Definitive radiotherapy without skull base surgery

	Sample size/Histology	RT dose	OS at 5 years	DSS at 5 years	DFS at 5 years	RFS at 5 years	Poor Prognosticators
Ballantyne et al.	80; Mostly SCC	Not clearly described	-	-	-	46%	-
Bourne et al.	13; SCC;	50-55Gy/20-25#	-	-	-	20%	-
Garcia Serra et al.	76; SCC or BCC	Median dose: 70 Gy/39# in either once-daily or twice-daily fractionation. 2 dimensional RT planning	-	-	-	50%	-
Lin et al.	44; SCC	Median dose 60Gy in 30 fractions at 5 fractions/week.	-	-	-	39%	Multiple cranial nerves; V1 and/or V2 nerve PNS worse than V3 or facial nerve
Balamucki et al.	65; SCC or BCC	Median dose: 74.9GGy/39# or 70.2Gy and interstitial implant; once daily or twice daily treatment	54%	64%	51%	-	Non-significant trend towards worse outcome for macroscopic/ central disease.

Abbreviations: SCC: Squamous cell carcinoma; PNS: Perineural spread; RT: Radiotherapy; OS: Overall survival; DSS: Disease specific survival; DFS: Disease free survival; RFS: Relapse free survival
